# Post-cholecystectomy Cystic Duct Stump Calculus With Cystic Duct Remnant-Common Bile Duct Fistula Causing Choledocholithiasis: A Report of a Rare Case

**DOI:** 10.7759/cureus.109126

**Published:** 2026-05-18

**Authors:** Md. Akbar Khan, Ambar R Shaikh, Vishwa P Jha, Anil Kumar

**Affiliations:** 1 Department of General Surgery, Madhubani Medical College, Madhubani, IND

**Keywords:** choledocholithiasis, cystic duct stump stone, post-cholecystectomy, recurrent biliary symptoms, t-tube drainage

## Abstract

Choledocholithiasis after cholecystectomy is a clinically significant cause of recurrent biliary symptoms and may result from retained stones, recurrent stone formation, or calculi within the cystic duct remnant. Although uncommon, cystic duct stump calculi can present several years after surgery and may be associated with chronic inflammation, adhesions, and, rarely, biliary fistula formation.

We report the case of a 48-year-old female with a history of open cholecystectomy who presented with recurrent right upper abdominal pain, intermittent jaundice, clay-colored stools, and high-colored urine. Magnetic resonance cholangiopancreatography (MRCP) revealed choledocholithiasis with proximal biliary dilatation, along with a cystic duct stump calculus. The patient was planned for choledochoduodenostomy; however, intraoperative findings included dense adhesions, a cystic duct remnant-common bile duct (CBD) fistula, and an impacted stump stone, making the planned procedure unsafe. The surgical approach was modified, and CBD exploration with stone extraction and T-tube drainage was performed. The postoperative course was uneventful, and a T-tube cholangiogram confirmed satisfactory ductal clearance. The patient remained asymptomatic on follow-up, with normalization of liver function tests.

This case highlights the importance of considering cystic duct stump pathology in post-cholecystectomy patients with recurrent biliary symptoms and underscores the need for individualized surgical decision-making in complex reoperative biliary surgery.

## Introduction

Choledocholithiasis occurring after cholecystectomy represents a diagnostically important and surgically relevant condition, especially in patients who continue to have recurrent biliary pain, jaundice, dyspeptic symptoms, or cholangitic episodes after presumed definitive gallbladder surgery [1,2]. The etiology in such patients may include residual common bile duct (CBD) stones missed at the time of the initial surgery, recurrent primary bile duct stone formation, migration of retained stones, or calculi lodged within a long cystic duct remnant or gallbladder stump [2,3]. Among these causes, cystic duct stump calculi are relatively uncommon and often underrecognized because the clinical presentation may be delayed by months or even years after the primary operation [3,4]. Patients may present with chronic right upper quadrant pain, intermittent obstructive jaundice, cholestatic liver enzyme abnormalities, recurrent cholangitis, or nonspecific upper abdominal symptoms, making diagnosis challenging in routine surgical practice [1,4].

With the wider availability of cross-sectional biliary imaging, especially magnetic resonance cholangiopancreatography (MRCP), the preoperative identification of retained ductal stones and remnant duct pathology has improved considerably [5]. MRCP is noninvasive and provides excellent delineation of the biliary tree, helping identify ductal dilatation, filling defects, residual cystic duct remnants, and associated pathology such as upstream biliary obstruction [5]. However, despite improved imaging, definitive management in complex post-cholecystectomy cases still frequently depends on operative exploration, particularly when endoscopic treatment is not feasible or when there is associated fistula formation, dense adhesions, or altered anatomy [4,6].

Biliary fistulae, including cystic duct remnant-CBD fistulae, develop as a consequence of chronic inflammation, adhesions, pressure necrosis, and longstanding stone disease [7]. Their coexistence with retained biliary calculi further increases operative difficulty and may substantially alter the planned surgical procedure. We present a case of choledocholithiasis after open cholecystectomy caused by an impacted cystic duct stump/gallbladder stump stone with an associated cystic duct remnant-CBD fistula, managed successfully with CBD exploration, stone extraction, and T-tube drainage.

## Case presentation

A 48-year-old female was admitted to Madhubani Medical College and Hospital with complaints of recurrent pain in the right upper abdomen for approximately six years. The pain had been intermittent in nature and had persisted since she underwent an open cholecystectomy seven years earlier. In addition to the chronic pain, she gave a history of episodic yellowish discoloration of the eyes for nearly six years, suggesting repeated biliary obstruction of varying severity. One week before admission, she developed clay-colored stools, and over the preceding five days, she noticed high-colored urine. She also had vomiting for three to four days before presentation. There was no mention of hematemesis, melena, abdominal distension, or altered bowel habits. Taken together, her symptoms suggested recurrent biliary obstruction with recent worsening.

On admission, routine laboratory investigations revealed a hemoglobin level of 9.1 g/dL, indicative of anemia, while the total leukocyte count (5330/mm³) and platelet count (282,000/mm³) were within normal limits. Biochemical parameters showed a total bilirubin of 1.58 mg/dL and direct bilirubin of 1.05 mg/dL, which were only mildly elevated; however, in the context of a documented history of intermittent jaundice, these findings were consistent with episodic biliary obstruction. Liver enzymes were deranged, with elevated transaminases (serum glutamic-oxaloacetic transaminase (SGOT), 111.62 U/L, and serum glutamic-pyruvic transaminase (SGPT), 69.6 U/L) and a raised alkaline phosphatase level of 323 U/L, suggesting a predominantly cholestatic pattern of liver injury. Renal function tests, serum electrolytes, total protein, and albumin levels were within normal limits, indicating preserved renal function and adequate nutritional and hepatic synthetic status. In view of the anemia, one unit of B-positive packed red blood cells was transfused preoperatively. Overall, the laboratory profile supported a diagnosis of recurrent biliary obstruction with preserved physiological reserve for surgical intervention (Table 1).

**Table 1:** Baseline laboratory investigations on admission. SGOT: serum glutamic-oxaloacetic transaminase (aspartate aminotransferase); SGPT: serum glutamic-pyruvic transaminase (alanine aminotransferase); Na⁺: sodium; K⁺: potassium; g/dL: grams per deciliter; mg/dL: milligrams per deciliter; U/L: units per liter; /mm³: per cubic millimeter

Parameter	Observed Value	Reference Range	Interpretation
Hemoglobin (g/dL)	9.1	12-16 (female)/13-17 (male)	Decreased - anemia
Total Leukocyte Count (/mm³)	5,330	4,000-11,000	Within normal limits
Platelet Count (/mm³)	282,000	150,000-450,000	Within normal limits
Total Bilirubin (mg/dL)	1.58	0.3-1.2	Mildly elevated
Direct Bilirubin (mg/dL)	1.05	0-0.3	Elevated
SGOT/AST (U/L)	111.62	5-40	Elevated
SGPT/ALT (U/L)	69.6	5-40	Elevated
Alkaline Phosphatase (U/L)	323	44-147	Markedly elevated
Renal Function Tests (Urea/Creatinine)	Within normal limits	Urea: 10-40 mg/dL; Creatinine: 0.6-1.2 mg/dL	Normal
Serum Electrolytes	Within normal limits	Na⁺: 135-145 mmol/L; K⁺: 3.5-5.0 mmol/L	Normal
Total Protein (g/dL)	Within normal limits	6.0-8.3	Normal
Serum Albumin (g/dL)	Within normal limits	3.5-5.0	Normal

According to the MRCP report, which was performed preoperatively, findings were consistent with choledocholithiasis with upstream biliary dilatation and a cystic duct stump calculus (Figure 1). The imaging demonstrated a T2 hypointense filling defect measuring approximately 1.4 cm within the distal CBD, associated with dilatation of the proximal CBD, common hepatic duct, right and left hepatic ducts, and intrahepatic biliary radicals, indicating significant biliary obstruction. In addition, a second T2 hypointense calculus of similar size was identified in the region of the cystic duct stump, suggesting remnant duct pathology following prior cholecystectomy. The overall radiological impression was choledocholithiasis with proximal biliary dilatation, in association with a cystic duct stump calculus. These findings were clinically important, as they provided a clear anatomical basis for the patient’s long-standing recurrent biliary symptoms and played a crucial role in guiding subsequent surgical planning.

**Figure 1:**
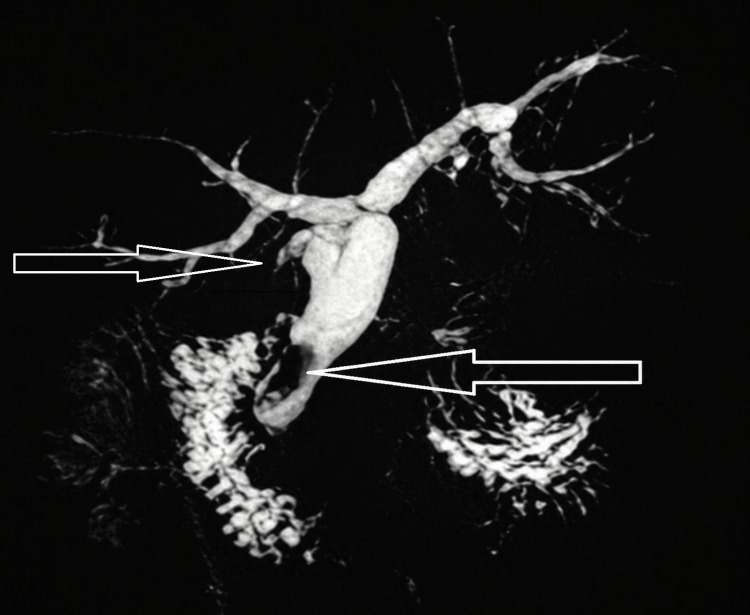
Magnetic resonance cholangiopancreatography (MRCP) demonstrating a distal common bile duct stone with proximal biliary dilatation (arrows indicating the site of the stone), along with a cystic duct stump calculus.

In view of the preoperative imaging findings and the patient’s history of recurrent obstructive biliary symptoms, surgical intervention was planned, with an initial intent to perform choledochoduodenostomy to address the ductal obstruction. However, upon entering the abdominal cavity, the operative field was found to be significantly distorted by dense adhesions, with bowel loops and omentum firmly plastered over the liver, indicating longstanding inflammatory changes and a technically challenging reoperative scenario. Careful and meticulous adhesiolysis was undertaken, during which a cystic duct remnant-CBD fistulous communication was identified, likely resulting from chronic inflammation and pressure effects of long-standing impacted calculi. Further exploration of the operative field revealed an impacted stone located within the gallbladder stump/cystic duct stump region, correlating with the preoperative MRCP findings. In view of the dense adhesions, altered biliary anatomy, and the presence of a fistulous tract, proceeding with the originally planned choledochoduodenostomy was deemed unsafe due to the high risk of complications. Consequently, the surgical strategy was judiciously modified, and the CBD was opened to allow direct exploration. The impacted calculus was carefully extracted from the stump region (Figure 2), ensuring adequate ductal clearance and addressing the primary source of obstruction. This intraoperative decision-making highlights the importance of flexibility and individualized surgical planning in complex reoperative biliary surgery.

**Figure 2:**
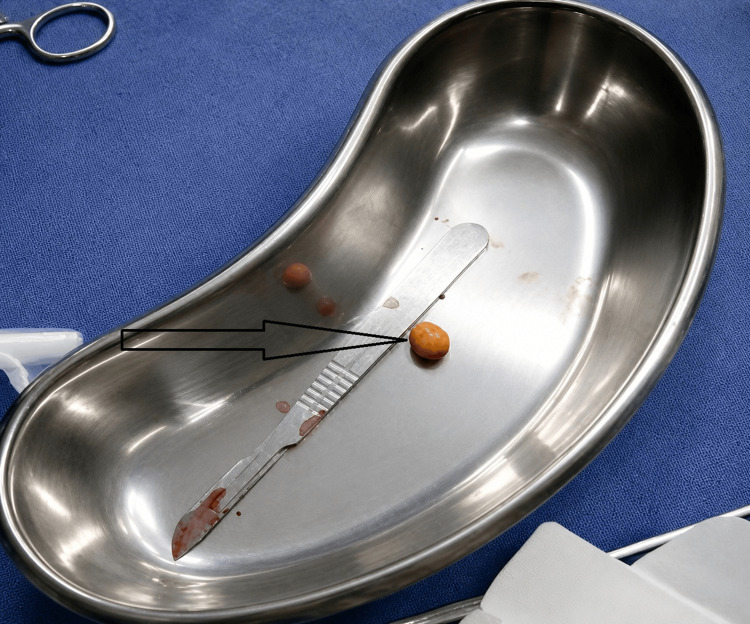
Intraoperative specimen demonstrating an extracted cystic duct stump stone placed on a surgical tray (arrow indicating the calculus).

After ensuring satisfactory ductal clearance following CBD exploration, a T-tube was inserted into the CBD and securely positioned to facilitate controlled external biliary drainage (Figure 3). The use of T-tube drainage was considered appropriate in this complex reoperative setting, as it provided decompression of the biliary system, allowed postoperative monitoring of bile output, and enabled access for cholangiographic evaluation to confirm ductal patency and exclude residual stones or leaks. Following placement of the T-tube, meticulous hemostasis was achieved, and the abdomen was closed in layers.

**Figure 3:**
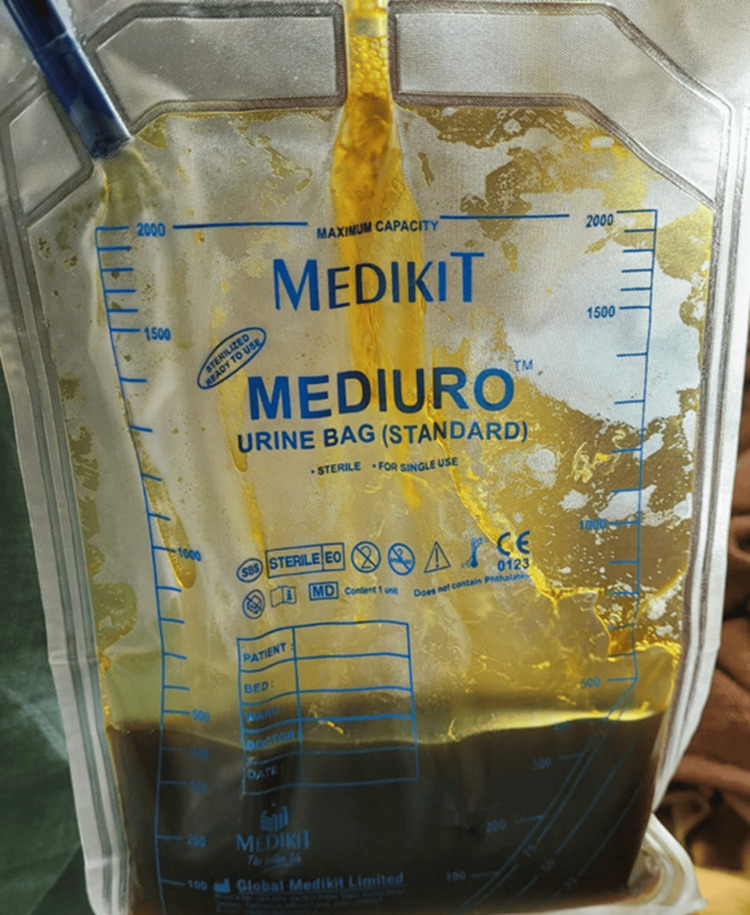
T-tube drainage.

Postoperatively, the patient was managed under close in-hospital monitoring, with regular assessment of vital parameters, abdominal examination, and T-tube output to ensure adequate biliary drainage. Her recovery remained clinically stable and uneventful, with no evidence of immediate postoperative complications such as bile leak, hemorrhage, sepsis, or features of persistent biliary obstruction. She was observed for a total duration of 10 days, during which appropriate supportive care, including analgesia, fluid management, and nutritional support, was provided, along with meticulous drain monitoring and serial clinical evaluations. Prior to discharge, a T-tube cholangiogram was performed to assess the integrity and patency of the biliary tree. The study demonstrated free and unobstructed flow of contrast into the duodenum, with no evidence of residual calculi, strictures, or contrast extravasation, thereby confirming successful ductal clearance and absence of leak (Figure 4). In view of her stable clinical condition and satisfactory cholangiographic findings, the patient was deemed fit for discharge with the T-tube in situ and was advised regular follow-up for subsequent management.

**Figure 4:**
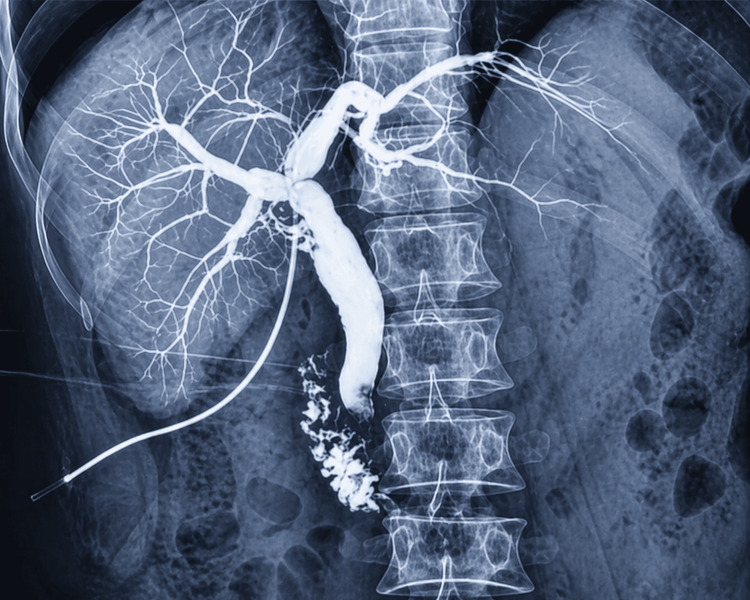
T-tube cholangiogram demonstrating the intrahepatic and extrahepatic biliary ducts, with contrast flow into the duodenum.

Since the patient was clinically stable and tolerating treatment well, she was discharged with the T-tube in situ and advised follow-up.

## Discussion

Choledocholithiasis after cholecystectomy continues to be an important clinical problem because the persistence or recurrence of biliary symptoms after prior gallbladder surgery is often assumed to have been definitively treated, which may delay further evaluation [1,2]. In many such patients, symptoms are due to retained CBD stones, recurrent primary bile duct calculi, or calculi in a residual cystic duct or gallbladder stump [2-4]. The present case is notable for the chronicity of symptoms, with right upper abdominal pain and intermittent jaundice continuing for several years after open cholecystectomy, ultimately found to be related to choledocholithiasis and a stump stone. This prolonged course is consistent with prior reports showing that remnant duct calculi can remain undiagnosed for years and may present with intermittent obstructive symptoms rather than dramatic acute cholangitis [3,4].

The cystic duct stump is an important, but sometimes overlooked, site of retained or recurrent calculi after cholecystectomy. A long remnant, incomplete excision, difficult Calot’s triangle dissection during the initial surgery, or subtotal cholecystectomy can contribute to persistence of stone-bearing tissue [3,4]. Stones in this region may produce a ball-valve type obstruction, intermittent biliary colic, or recurrent inflammatory episodes. Because the symptoms mimic other causes of biliary obstruction, a high index of suspicion is required, especially when the patient has a convincing post-cholecystectomy biliary history. In our patient, the preoperative MRCP was particularly valuable because it identified not only choledocholithiasis with proximal biliary dilatation, but also a stump calculus, thereby raising suspicion for a more complex biliary pathology before re-exploration [5].

MRCP has become one of the most useful investigations in such patients because it is noninvasive and accurately delineates the biliary anatomy [5]. In post-cholecystectomy patients, it can demonstrate bile duct stones, ductal dilatation, remnant cystic duct pathology, and other structural abnormalities. This is particularly relevant in patients with previous open surgery, where secondary operative fields are often complicated by dense adhesions and altered tissue planes. Although endoscopic retrograde cholangiopancreatography may be both diagnostic and therapeutic for many bile duct stones, not all cases are suitable for endoscopic management, especially when stump anatomy is unusual, stones are impacted, or there is associated fistulous disease [4,6].

An additional important aspect of the present case was the intraoperative finding of a cystic duct remnant-CBD fistula. Fistulae usually arise in the setting of chronic gallstone disease and repeated inflammation, where pressure from impacted stones and persistent local inflammatory reaction ultimately erode into adjacent bowel, most commonly the duodenum [7]. The discovery of such a fistula explained the dense adhesions and difficult surgical field in our patient. It also significantly influenced intraoperative decision-making. While choledochoduodenostomy had been planned preoperatively, the presence of adhesions, a fistulous tract, and distorted anatomy made this option unsafe. This highlights a central principle of hepatobiliary surgery: the need to modify the operative plan according to the actual anatomical and inflammatory findings encountered during surgery, rather than rigidly adhering to the preoperative plan.

In this setting, CBD exploration with stone extraction and T-tube drainage represented a prudent and effective alternative. T-tube drainage has historically played an important role after bile duct exploration, particularly in difficult cases, because it allows decompression of the biliary tree, access for postoperative cholangiography, and time for edema to settle before complete internal biliary flow is relied upon [6,8]. Although primary closure of the CBD is increasingly practiced in selected patients, T-tube insertion remains useful in reoperative biliary surgery, in cases of uncertain ductal clearance, inflamed tissues, and anatomically complex situations [8]. In our patient, this approach allowed safe postoperative monitoring and confirmation of ductal patency through cholangiography before tube removal.

The postoperative course in the present case further supports the effectiveness of this management strategy. The patient recovered without major complications, had a normal T-tube cholangiogram on postoperative day 10, tolerated subsequent clamping and unclamping trials, and remained asymptomatic after tube removal, with normalization of liver function tests. This favorable outcome emphasizes that even in complex post-cholecystectomy biliary disease with fistulous pathology, satisfactory results can be achieved when surgery is individualized, careful ductal clearance is ensured, and staged postoperative assessment is used.

From a practical clinical standpoint, this case underlines several important messages. First, recurrent right upper quadrant pain, jaundice, clay-colored stools, and high-colored urine in a patient with prior cholecystectomy should prompt renewed evaluation for biliary obstruction rather than premature attribution of symptoms to nonspecific causes [1,2]. Second, stump calculi should remain in the differential diagnosis, especially when symptoms persist for years after surgery [3,4]. Third, MRCP is extremely helpful in defining biliary anatomy and planning intervention [5]. Finally, intraoperative flexibility is essential in reoperative biliary surgery, and T-tube drainage still has a relevant role in selected complex cases [6,8].

## Conclusions

Choledocholithiasis occurring after cholecystectomy should be carefully evaluated in patients presenting with recurrent biliary symptoms, even many years after the initial surgery. Cystic duct stump or gallbladder remnant calculi, although uncommon, represent an important and often underrecognized cause of persistent or recurrent obstruction, and may be associated with complex pathology such as fistulous communication. MRCP plays a crucial role in preoperative diagnosis and anatomical delineation; however, definitive management often depends on intraoperative findings. This case emphasizes the importance of maintaining a high index of suspicion for remnant biliary pathology, the need for flexibility in surgical planning, and the continued relevance of CBD exploration with T-tube drainage in difficult reoperative settings to achieve favorable outcomes.
